# The comparative aspects of hystricomorph subplacenta: potential endocrine organ

**DOI:** 10.1186/s40850-021-00074-w

**Published:** 2021-05-19

**Authors:** Maria Angelica Miglino, Gustavo de Sá Schiavo Matias, Nathia Nathaly Rigoglio, Jessica Borghesi, Taís Harumi de Castro Sasahara, Maria Josephina Illera del Portal, Juan Carlos Illera del Portal, Gema Silván Granado, Sara Cristina Caceres Ramos, Moacir Franco de Oliveira, Alan James Conley

**Affiliations:** 1 Department of Surgery, School of Veterinary Medicine and Animal Science (FMVZ-USP), University of São Paulo- SP, Ave. Prof. Dr. Orlando Marques de Paiva, 87, São Paulo, 05508270 São Paulo Brazil; 2 Department of Animal Physiology, School of Veterinary Medicine, Complutense University of Madrid (UCM), Madrid, Spain; 3 Department of Animal Science, Federal University of the Semi-Arid Region (UFERSA), Mossoro, Brazil; 4 Department of Population Health and Reproduction, School of Veterinary Medicine School of Veterinary Medicine, University of California UC-Davis, 3223 VM3B, Sacramento, California USA

**Keywords:** Rodents, Placentation, Hormones, Capybara, Agouti

## Abstract

**Background:**

The placenta of hystricomorph rodents, lagomorphs and some primates includes an unusual structure, termed a subplacenta, which essentially consists of trophoblastic cells located deep to the central implantation site within the area of decidualization. It has been suggested that the subplacenta is functionally important, although considerable controversy remains on the issue. In this context, our objective was to compare the architecture and structure of the subplacentas of different hystricomorph species, to investigate the possibility that it is active in hormone synthesis.

**Methods:**

In total, the placentas of 3 capybaras (*Hydrochaeris hydrochaeris*), 2 pacas (*Agouti paca*), 5 agoutis (*Dasyprocta leporina*), 5 rock cavies (*Kerodon rupestris*) and 3 guinea pigs (*Cavia porcellus*) at different stages of pregnancy (early, middle and near term) were used for gross and microscopic examination. This included the preparation of latex injection casts, immunohistochemistry for steroidogenic enzymes, scanning and transmission electron microscopy. Tissue steroid concentrations were also determined.

**Results:**

The gross morphology and microvascular arrangement of the subplacentas were similar among the hystricomorphs studied including ultra-structural verification of cytotrophoblast and syncytiotrophoblast in all species. In guinea pigs, trophoblast cells exhibited characteristics consistent with intense metabolic and secretory activity in general. However, immuno-histochemical evidence also indicated that subplacental trophoblast expressed key steroidogenic enzymes, mainly in the chorionic villus region, consistent with tissue steroid concentrations.

**Conclusions:**

The subplacentas within placentas of hystricomorph rodent species are structurally similar and, in guinea pigs, have potential for steroid hormone secretion from, at least the early stages of pregnancy.

## Background

The success of viviparity in eutherian mammals is dependent on the establishment of a fully functional chorioallantoic placenta in order to sustain successful embryonic and fetal development. In this context, placental development from trophoblast is mediated in part by interactions between maternal endometrial and extra-embryonic tissues during the implantation process [1–5]. The placenta is responsible for maternal-fetal metabolic exchange supporting the nutrition and respiration of, and excretion by, the fetus [6–8] and in some species, it elaborates hormones providing endocrine support for the pregnancy [9]. The structures associated with the placenta are complex and, depending on species, may consist of the amnion, chorion, allantois, yolk sac, and decidual elements [10–13]. In addition, these extra-embryonic membranes have substantive importance in ontogeny.

Some species within the Hystricomorph and Lagomorph sub-orders of the Rodentia have a specialized structure within their placentas, called a subplacenta [14–18], that is unusual among mammals. The structure is represented by zone of chorionic ectoderm, which is located in the central excavation of the placenta and is limited to the area of decidualization [19]. The functions of this placental specialization remain unclear [1]. While some have speculated that the subplacenta might play an endocrine role, possibly gonadotrophin secretion [15, 20, 21], no definitive evidence exists to date. Uptake of histotroph [20, 22] or lactogen secretion [21] have also been suggested as possible functions. However, according to [23] none of these hypotheses has been resolved to a sufficient degree.

It is presumed that the subplacenta plays an important, specialized role during pregnancy in hystricomorph rodents [18, 24–26]. However, despite the fact that this structure has been investigated in numerous morphological studies, there is a dearth of information in the literature in regard to the functions of this unusual placental structure. Therefore, the aim of this study was to describe and compare the subplacentas from different hystricomorph species using morphological techniques. In addition, other approaches including latex injections, immunohistochemistry, scanning and transmission electron microscopy and analysis of tissue steroid concentrations were also employed in order to determine if hormone synthesis was likely. These new data were discussed along with existing literature to better define functional correlates that might provide insight into the evolution of the subplacenta of hystricomorph rodents.

## Methods

### Animals and gross morphology

In total, placentae were analyzed from 3 capybaras (*Hydrochaeris hydrochaeris*), 2 pacas (*Agouti paca*), 5 agoutis (*Dasyprocta leporina*), 5 rock cavies (*Kerodon rupestris*), and 3 guinea pigs (*Cavia porcellus*) in different stages of pregnancy (early, middle and near term). The samples were collected from multiple mothers. The guinea pig pregnancy age estimation was performed based in [27, 28]. The animals were submitted to vaginal cytology to detect the onset of pregnancy, and ultrasound examination to monitor gestational development. The placentas were recovered after specific days of pregnancy (Table 1) and the euthanasia was carry out by ketamine (300 mg/kg) and xylazine (30 mg/kg) overdoses by intraperitonial administration. Samples were obtained from the Center for Experimental Breeding of Capybara, at São Paulo State University, Araçatuba-SP; Center for Experimental Breeding of Paca, at São Paulo State University, Jaboticabal-SP; Center for Experimental Breeding of Agouti, at Federal University of Piaui, Teresina-PI; and Center for the Breeding of Wild Animals, at Federal Rural do Semi-Árido University, Mossoró-RN, Brazil.

**Table 1:** Summary of species and the different techniques utilized on the tissue samples collected

Species	NS	TG	GM	L I	IM	SEM
capybara *(H. hydrochaeris)* *(150 days)*	3	end of gestation (90–140 days)	X	X		X
paca *(A. paca)* *(150 days)*	2	middle third of gestation (45–70 days)	X	X		
agouti *(D. leporine)* *(106 days)*	5	middle of gestation (35–50 days)	X	X		X
rock cavy *(K. rupestris)* *(76 days)*	5	end of gestation (60–75 days)	X	X		
guinea pig *(C. porcellus)* *(72 days)*	3	early (around 10 days), middle (around 35 days) and late (around 70 days) gestation	X		X	

*TG* Time of gestation, *GM* Gross Morphology, *LI* Latex Injection, *IM* Immunohistochemistry, *SEM* Scanning Electron Microscopy

Material for data analysis was collected following primary fixation with 4 % paraformaldehyde injected in situ via the maternal and fetal systems for further analysis and samples preservation. (Table 1). Gross aspects of the placentae were photographed, and schematic diagrams were drawn for documentation.

### Latex injections

As a further aid to understanding vessel distribution, some placentae in early/middle gestation were injected with coloured latex (Neoprene 650, DuPont, Brazil; Latex Stain, Suvinil, Glassurit do Brazil S/A, São Bernardo do Campo, S.P., Brazil). Different colours were injected in a uterine vein, a uterine artery, an umbilical artery and the umbilical vein. The placentae were then immersion fixed in 10 % formalin, dissected and analyzed.

### Microscopic anatomy

Preparations of histological sections for light microscopy were performed at the University of São Paulo’s School of Veterinary Medicine and Animal Science. Fragments of the placentas collected at different stages of gestation were fixed in 4 % paraformaldehyde solution. After fixation the material was washed in phosphate–buffered saline (PBS), followed by dehydration in a series of increasing concentrations of ethanol (from 70 to 100 %), followed by diaphanization in xylol and paraffin embedding (Histosec) [29]. Paraffin blocks were sectioned at 5 μm in an automatic microtome (Leica, RM2165, Germany). Sections were left to adhere overnight to glass slides in an incubator at 40 °C. After being deparaffinized, the sections were stained with hematoxylin and eosin, as well as toluidine blue using routine methods.

### Immunohistochemistry (IM)

Tissues collected for immunohistochemistry were immersion fixed in 4 % paraformaldehyde in 70 mM phosphate buffer for 48 h, and then washed in cold PBS for 72 h. The samples were embedded in paraffin, sectioned to 5 μm in a microtome (Leica RM2265) and transferred to silanized slides (Sigma, # p8920). The sections were rehydrated and washed in citrate buffer (1.83 mM citric acid hydrate and 8.9 mM tribasic sodium citrate, pH 6.0) for antigen recovery. Endogenous peroxidase was blocked with 3 % hydrogen peroxide diluted in distilled water for 30 min in a darkroom. Tissue sections were blocked with 2 % bovine serum albumin (BSA) diluted in PBS for 30 min. Primary antibodies: cytokeratin (catalogue number RGE53, Novus, 1:100), vimentin (GTX35160, GeneTex, 1:100), 17β-hydroxysteroid dehydrogenase (17β-HSD, abcam, catalogue number ab97971, 1:100), 3β-hydroxysteroid dehydrogenase (3β-HSD, Invitrogen, catalogue number FDO66Q 1:100), and aromatase cytochrome P450 (aromatase, Invitrogen, catalogue number PA1-21398 1:100) were incubated overnight in a dark, humid chamber at 4 °C. The reaction was revealed using Dako Advance HRP (Dako, catalogue number K6068) and stained with DAB (Dako, catalogue number K3468) according to the manufacturer’s instructions, and the slides were counterstained with hematoxylin. Between each step prior to antibody incubation, slides were rinsed in PBS containing 0.2 % BSA. Negative controls included no primary antiserum.

Slides were then mounted, viewed, and photographed on the Nikon Eclipse 80I microscope.

### Scanning Electron Microscopy (SEM)

To study the microvasculature, term placentae were injected with Mercox™ CL-2R resin (Okenshoji Co., Ltd, Tokyo, Japan) [30]. The procedures were similar to those applied by others to a wide range of placental types [31, 32]. A maternal and/or fetal vessel was cannulated and the resin injected under gentle pressure. Tissues were digested by immersion of the preparation in several changes of 20 % NaOH solution at 50–60 °C. The casts were rinsed thoroughly in distilled water, dried in an oven at 37 °C, and stored in 20 % gelatin at 4 °C. For scanning electron microscopy, pieces of the casts were rinsed in distilled water to remove the gelatin, dried, and mounted on stubs with conductive carbon cement (Neubauer, Münster, Germany). They were then coated with gold using a sputter coater (Model K550, Emitech Products Inc., Houston TX, USA) and examined in a scanning electron microscope (Model 435 VP, Leo Electron Microscopy Ltd, Cambridge, UK).

### Tissue steroid concentrations

Fragments of guinea pig placental tissue were collected at different stages of gestation (middle and late). Samples of subplacenta and labyrinthine zones were frozen at -20 °C for hormonal analysis. Steroid hormone concentration were measured using enzyme-immunoassays (EIA), previously validated by [33]. Steroid hormones were detected using specific primary antisera (estradiol, E2: C6E91; testosterone, T: R156 and progesterone, P4: C914) developed in the Department of Animal Physiology (UCM, Spain) and the Clinical Endocrinology Laboratory (University of California, Davis).

## Results

### Morphology and immuno-histochemistry

**Fig. 1:**
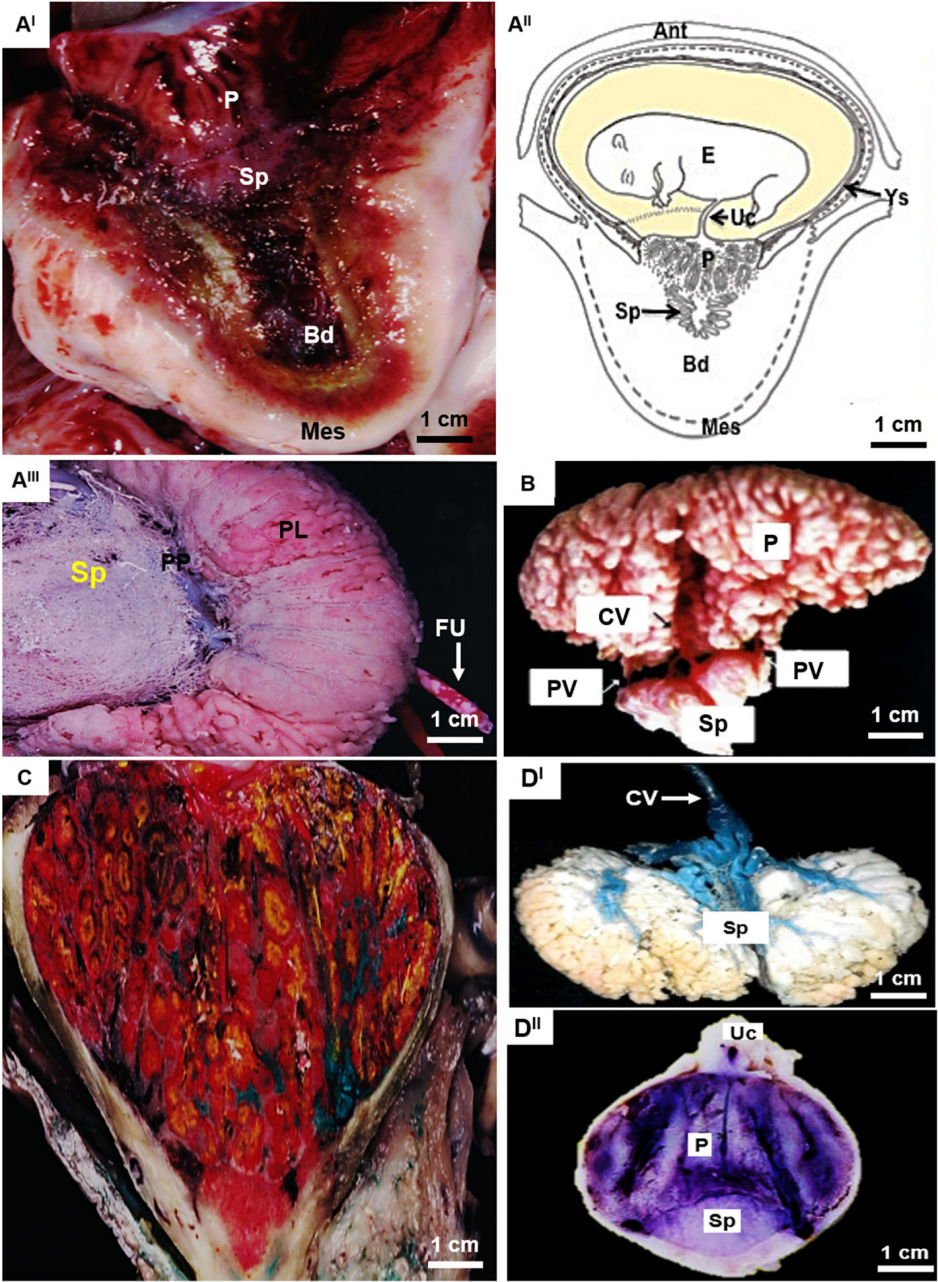
Gross morphology and microvascular architecture of the hystricomorph subplacenta. **A**^**I**^** – A**^**III**^ Capybara (*Hydrochaeris hydrochaeris*) around 90–140 days of gestation. **A**^**I**^ Sagittal section through the placenta showing the relationship among the chorioallantoic placenta (P), the subplacenta (Sp) and the basal deciduous (Bd) and the mesometrial side (Mes); **A**^**II**^ Schematic drawing illustrating the relationship between the fetus (F), umbilical cord (Uc), yolk sac (Ys), Sp and Bd, and anti-mesometrial (Ant) of the uterus; **A**^**III**^ Corrosion model of the capybara which the different types of microvascularization of the placental lobes (PL) and Sp are observed. Placental vessels were injected with methacrylate and subjected to the corroding process. The relationship between Sp microvessels and larger vessels is verified (arrow), which possibly carry products produced by the subplacenta during this gestation phase - PP (placental peduncle) FU (umbilical funiculum). **B** Rock cavy (*Kerodon rupestris*) around 76 days: Placenta injected with methacrylate submitted to corrosion showing the central vessels (CV) and peripheral vessels (PV) in relation to the chorioallantoic placenta (P) and Sp. **C** Paca (*Agouti paca*) around 45–70 days: In **C**, the paca placenta injected with latex, the maternal vein injected with green, maternal artery with white, fetal vein with yellow and fetal artery with red. Note, the Sp appeared to have regressed in size at term compared with mid-gestation. **D**^**I**^** – D**^**II**^ Agouti (*Dasyprocta leporine*) around 35–50 days: In **D**^**I**^, model of microvascularization of the agouti subplacenta, showing the central vessels injected in blue latex and the subplacenta in white latex. **D**^**II**^ Showing the P and Sp and also the umbilical cord (Uc)

The gross morphology and microvascular arrangements of the subplacenta of these hystricomorph rodents were observed during pregnancy (Fig. 1A-D). The subplacentas in pacas appeared to regress in size from the middle of pregnancy to full term (Fig. 1C) and the subplacenta of the rock cavy was entirely absent by full term (Fig. 1D^II^). However, a major gross vasculature structure was formed by maternal veins (Fig. 1D^II^).

**Fig. 2:**
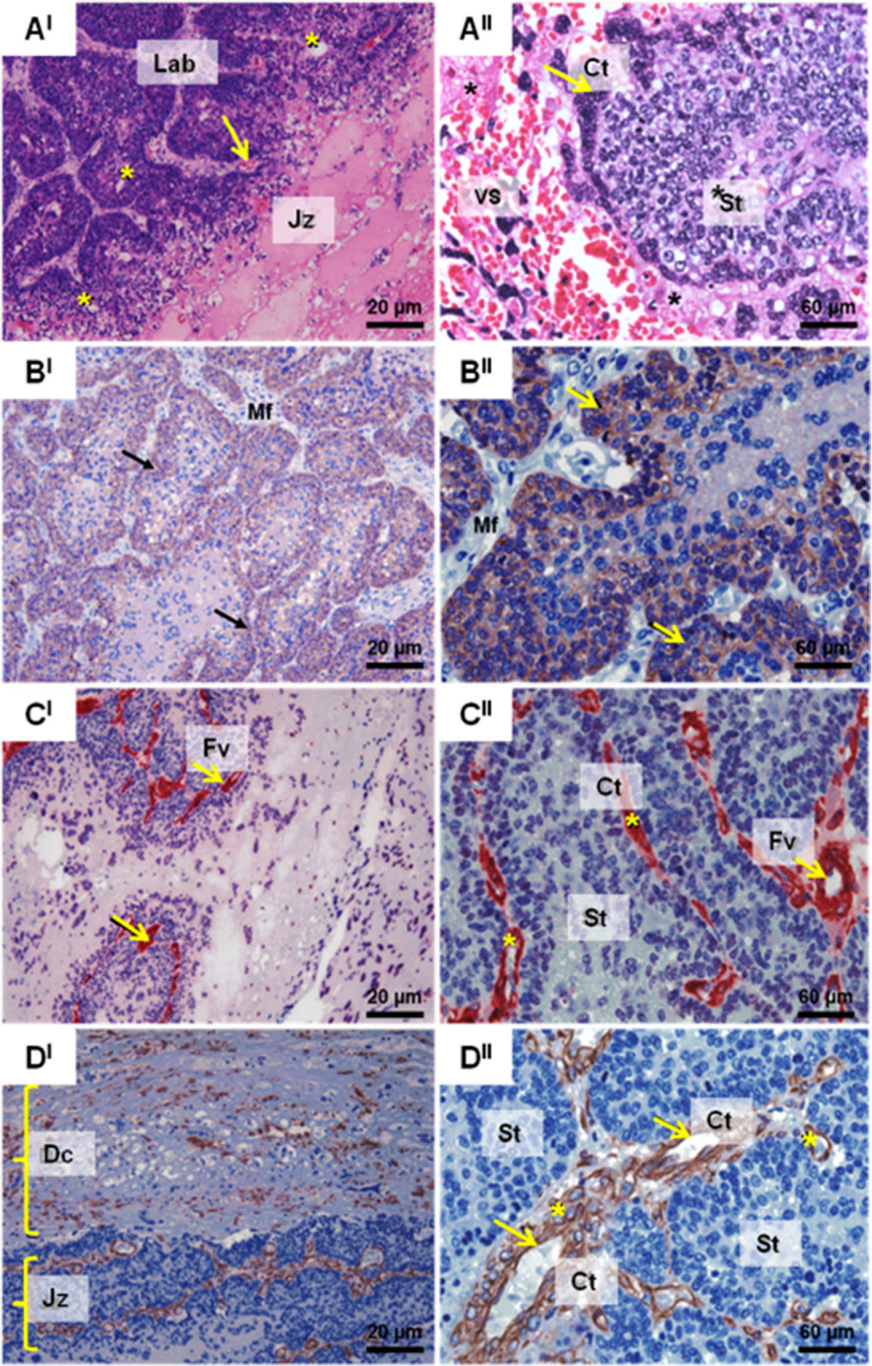
Capybara subplacenta (*H. hydrochaeris*) around 90–140 days of gestation. **A**^**I**^ Section through the subplacenta showing the junctional zone (Jz) with fetal (arrow) and maternal (*) vessels in the labyrinth layer. **A**^**II**^ Subplacenta showing the cytotrophoblast (Ct, arrow) interspersed by the cells of the syncytiotrophoblast (*St) surrounded by vessels (vs.). **B**^**I**^ Subplacenta capybara cytokeratin 20X. Observe a positive reaction present in giant trophoblastic cells in the cytotrophoblast region (arrow) and negative reaction in the fetal mesenchyme. **B**^**II**^ Subplacenta capybara cytokeratin 60X. Observe a positive reaction present in giant trophoblastic cells in the cytotrophoblast region (arrow) and negative reaction in the fetal mesenchyme (Mf). **C**^**I**^ Subplacenta capybara actin 20X. **C**^**II**^ Subplacenta capybara actin 60X. Positive marking on the muscular layer of the wall of fetal vessels (Fv) of Capybara Subplacenta (arrow) and cytotrophoblast (Ct-asterisk) cells and negative reaction in the region of the syncytiotrophoblast (St). **D**^**I**^ Subplacenta capybara vimentin 20X - Observe the deciduous region (Dc) of the placenta of the capybara and junctional zone (Jz). **D**^**II**^ Subplacenta capybara vimentin 60X - Observe positive marking for vimentin in the wall of vessels (arrow) and giant cells (asterisks) surrounded by cytotrophoblast (Ct) cells around the vessels, interspersed by the syncytiotrophoblast (St) region of the Capybara placenta

Fetal and maternal blood vessels were prominent in the labyrinth layer and near the junctional zone of the capybara subplacenta (Fig. 2A). Giant cytotrophoblast cells were interspersed with syncytotroblast cells, which were in turn surrounded by vessels (Fig. 2A^II^). The giant cells of the cytotrophoblastic region showed a positive reaction to cytokeratin, however there was no immuno-staining in the fetal mesenchyme (Fig. 2B^I − II^). There was positive actin labeling in the cells of the muscle layer of the fetal vessels, but no evidence of actin immuno-staining in the syncytotrophlast region (Fig. 2C^I − II^). Vimentin-positive labeling was observed on vessel walls and giant cells (Fig. 2D^I − II^).

**Fig. 3:**
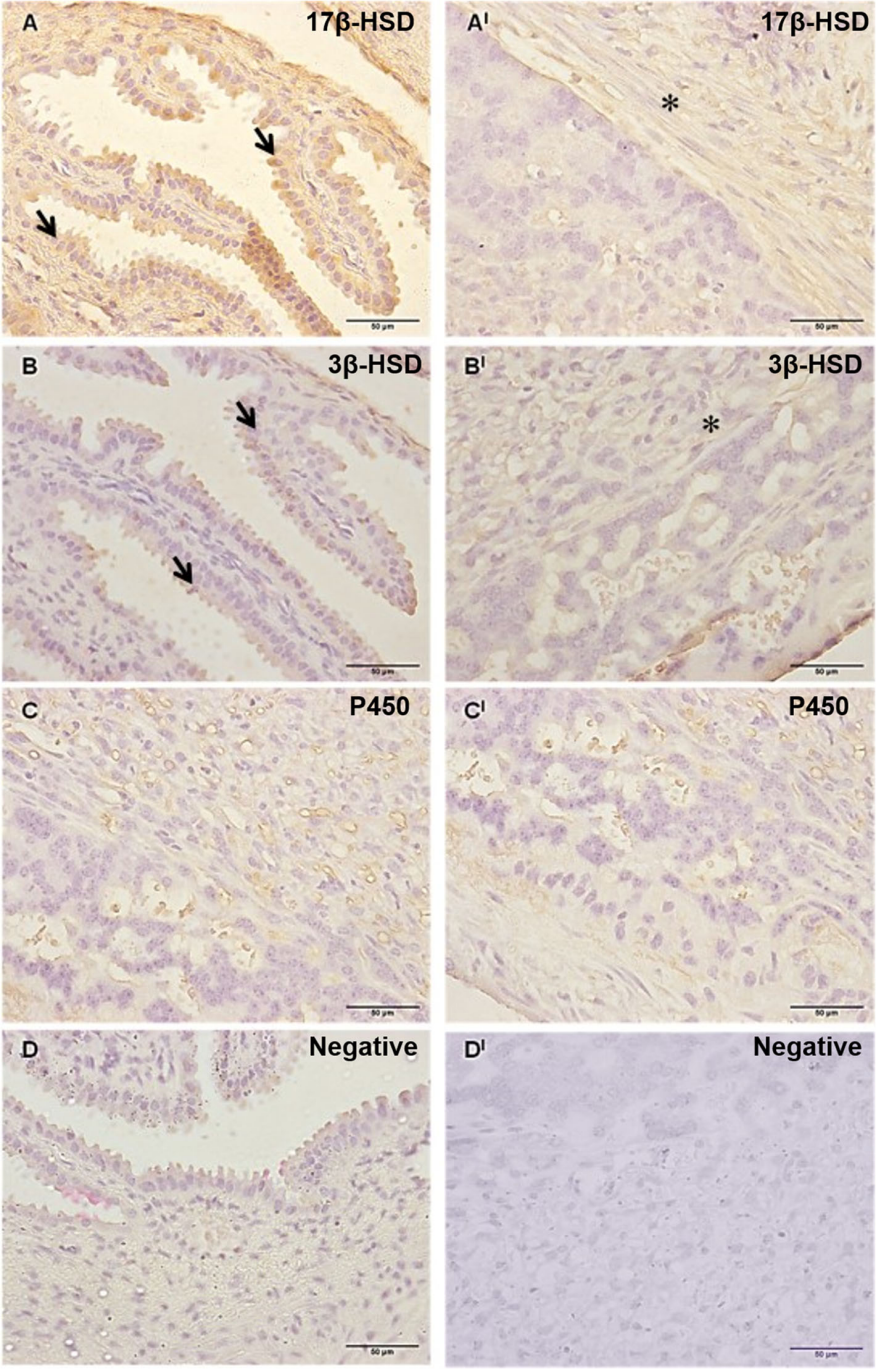
Immunohistochemistry (IM) from guinea pig *(C. porcellus)* subplacenta (early gestation). In **A, A**^**I**^and **B, B**^**I**^, positive labeling of 17β-HSD and 3β-HSD respectively in the villi (arrows) and in the labyrinth region (asterisks). In **C, C**^**I**^ positive expression of aromatase (P450). In **D, D**^**I**^, no labeling was observed in the negative control

In guinea pig placentae, there was positive immuno-reactivity consistent with steroidogenesis, mainly in the region of chorionic villi and, in the labyrinth region, only in the blood vessel region. Among the enzymes tested, 17β-HSD expression was especially evident in the trophoblastic cells of the villi (Fig. 3A, A^I^). Evidence of expression of 3β-HSD was also evident in the same region but was not as extensive expression (Fig. 3B, B^I^). The expression of aromatase (P450) was positive around the peripheral membrane protein of the labyrinth region (Fig. 3C, C^I^). No labeling was observed in the absence of the primary antisera, the negative controls (Fig. 3D, D^I^), in any of the sections.

### Microvascular arrangements

**Fig. 4:**
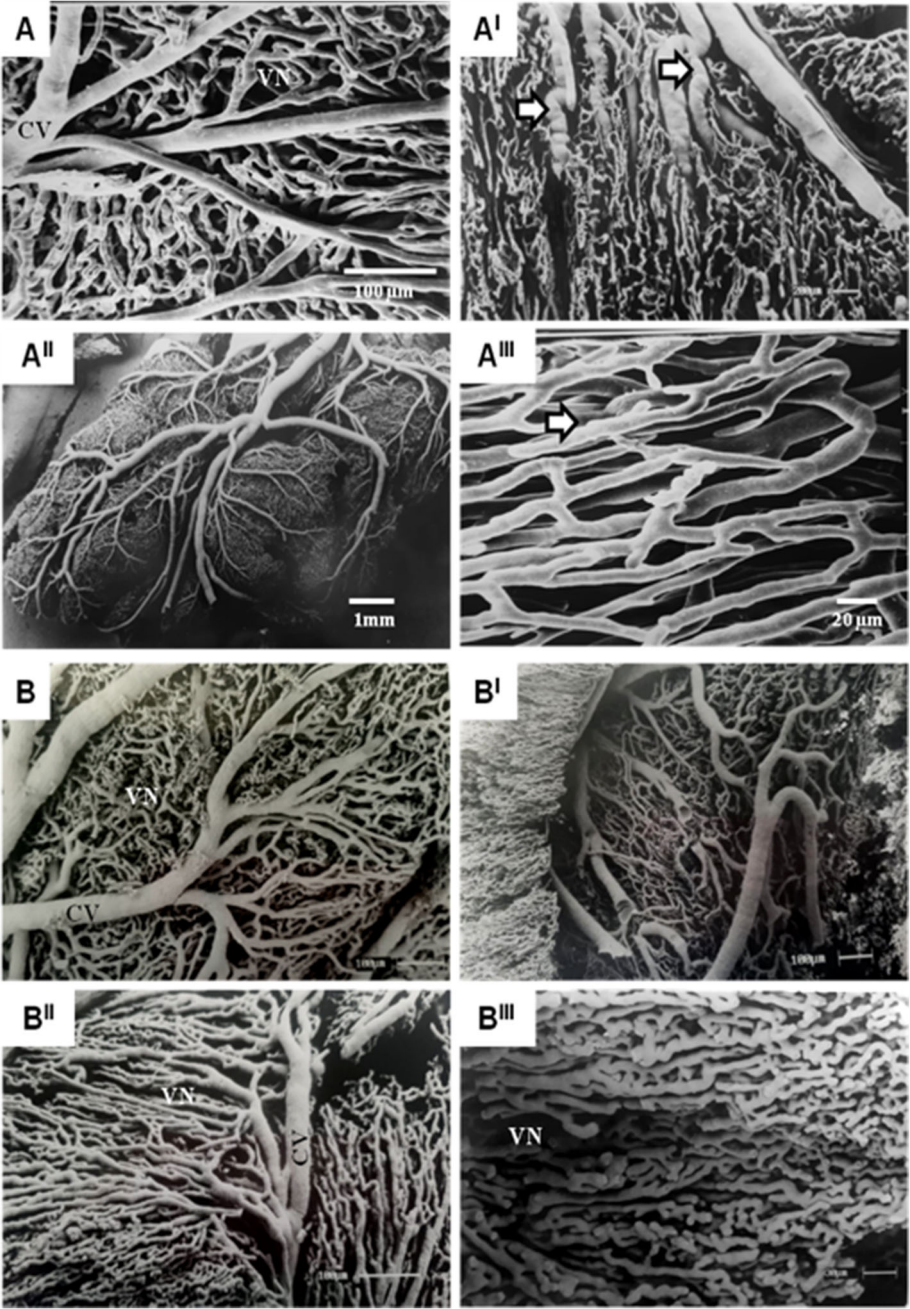
Scanning Electron Microscope (SEM) images of Capybara (*H. hydrochaeris*) subplacenta vascular molds around 90–140 days (**A-A**^**III**^) and Agouti (*D. leporine*) around 35–50 days (**B-B**^**III**^). In mid to late gestation, the subplacenta is supplied exclusively by fetal vessels. A large branch of the umbilical artery follows the central band of fetal mesenchyme to the base of the main placenta and then large central vessel (CV) insert to supply the subplacenta. The subplacental vessels pursue a tortuous course forming a vascular network (VN) with sinusoidal dilatations and constrictions (full arrow) in both species at different stages of pregnancy, which demonstrates that the vascular supply remains for the transportation of the products necessary for organ development

Vascular molds examined by the scanning electron microscopy technique demonstrated evidence of larger branches supplying the subplacenta. The division of these larger branches allows the branching of smaller vessels, which become tortuous with sinus dilations and constrictions in the vessel lumen as they course through the tissue (Fig. 4).

### Ultra-structure

**Fig. 5:**
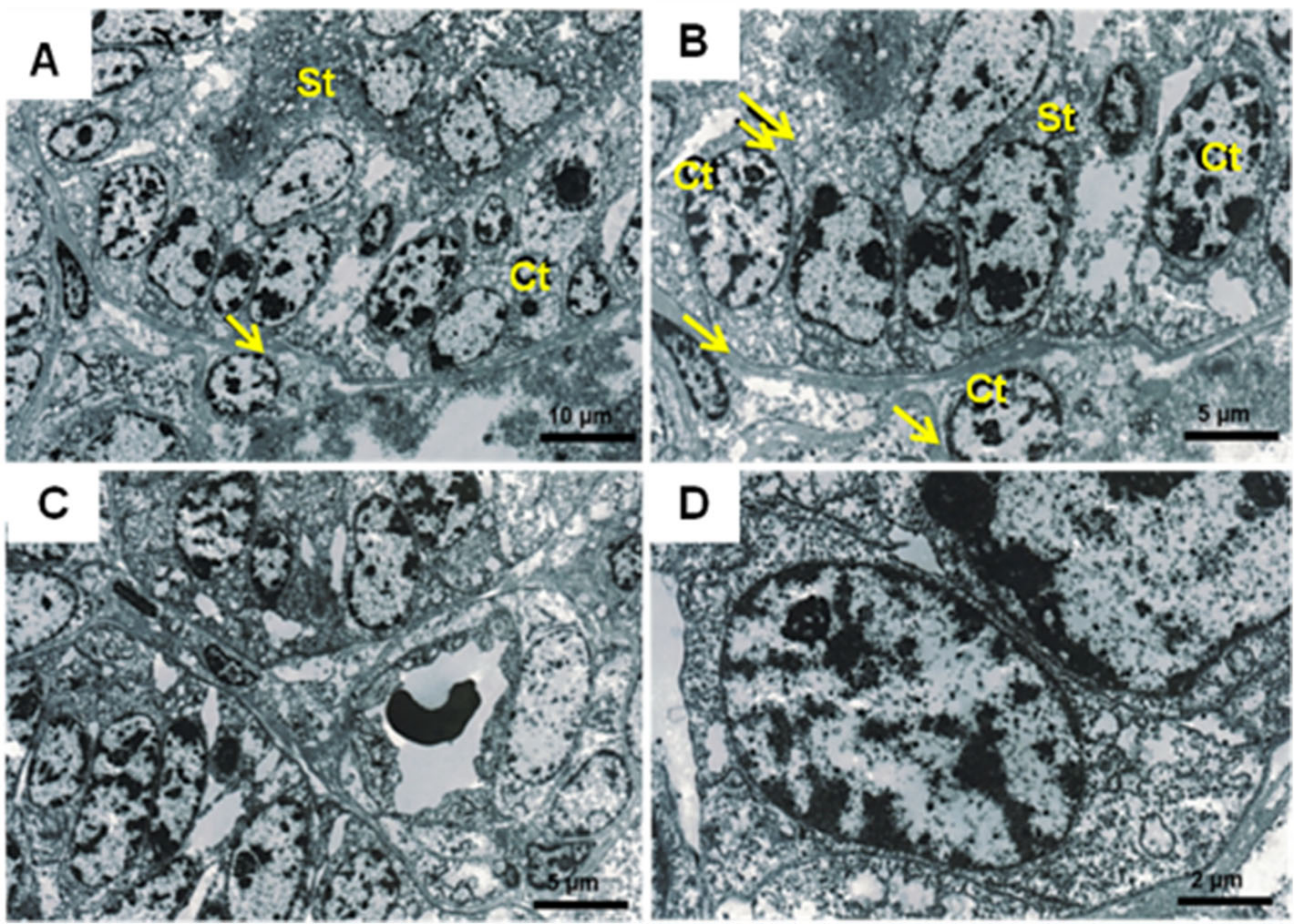
Transmission electron microscope (TEM) images of Subplacenta of the Paca (**a-d**). Observe the presence of cytotrophoblast (Ct) giant cells interspersed with the syncytiotrophoblast (St), basal lamina (arrow) and intercellular spaces (double arrow) are present

In capybara the nuclei of the cytotrophoblast cells were large in relation to the amount of cytoplasm and nucleoli were prominent (Fig. 5). The basal membrane of the cytotrophoblast layer was seen to be in contact with the connective tissue lamellae and often close to the fetal vessels. The syncytiotrophoblast enclosed many lacunae into which microvilli projected from the plasma membrane and, as gestation advanced, these lacunae formed an extensive inter-connected system.

### Tissue steroid content

To verify the capacity for hormonal production by the subplacenta, samples of guinea pig subplacenta were analyzed for progesterone, testosterone and estradiol content and compared with the labyrinth region. In general, for all steroids, samples from mid-gestation had higher steroid concentrations when compared to samples collected from late gestation. In addition, the subplacenta region showed higher hormone levels than labyrinthine samples (Table 2).

**Table 2:** Median concentrations (ng/mg of tissue) of progesterone, testosterone and estradiol extracted and measured in 3 differentes samples from guinea pig labyrinth and subplacenta tissue samples

	Progesterone		Testosterone		Estradiol	
	Middle	Late	Middle	Late	Middle	Late
**Labyrinth**	15.15 ± 1.74	8.87 ± 3.66	10.01 ± 4.09	5.52 ± 1.82	0.71 ± 0.05	0.55 ± 0.05
**Subplacenta**	24.88 ± 5.19	16.77 ± 2.87	15.59 ± 2.99	11.14 ± 0.17	0.95 ± 0.09	0.71 ± 0.02

## Discussion

The subplacenta, as a structure found only on hystricomorph rodents, lagomorphs, and some primates, has attracted interest, especially in terms of comparative morphology. However, to the best of our knowledge, no studies have yet established an endocrine function for the subplacenta during conceptus development. The results of early studies investigating the potential for progesterone synthesis by the guinea pig placenta indicated that the spongy trophoblast was a likely source [34, 35]. However, these investigators did not examine the subplacenta. To explore the potential for endocrine function, we investigated the comparative morphology of the subplacenta of several Hystricomorph rodents, with a particular focus on cell structures typical of secretory activities, as well as the presence of steroid hormones and enzymes involved in their synthesis.

The subplacenta has been described in several species among the Hystricomorph rodents [1, 14, 15, 24, 25, 36–40]. In these species, the development of subplacenta is marked by an increase in the thickness of the cytotrophoblast lamellae forming the floor of the central excavation. This occurs during the early development of the viscacha (*Lagostomus maximus*) subplacenta [41] and was observed at later stages in the chinchilla (*Chinchilla lanigera*) and coypu (*Myocastor coypus*) [42]. All caviomorph rodents exhibit a subplacenta located deep within the disc of the main placenta [43], which appears at the beginning of pregnancy and regresses during the later stages of development. Certain aspects related to the vascularization of the subplacenta remain to be defined. In nutria (*Myocastor coypus*) [42], the majority of maternal arteries are diverted from the subplacenta. Some of these arteries were observed to traverse the lobes of the subplacenta, but most were located outside of the subplacental area and thus between the adjacent lobes of the chorioallantoic placenta toward the central zone of each lobe. However, previous studies of rodent placentation [23, 43, 44] have provided scant evidence of the function of the subplacenta, suggesting only that it is a region contributing to cytotrophoblast growth and possible endocrine secretion.

Prior indirect evidence supports a possible endocrine role for the subplacenta. Recent work resulted in the creation of a library of differentially expressed genes (DEGs) from the subplacenta of the beaver (*Castor fiber*) carrying twin versus triplet fetuses. The transcripts identified were consistent with functions related to cellular processes, biological regulation, response to stimuli and metabolism but, compared to other parts of the placenta or even compared to other species, there appeared to be little difference in functional terms [45]. The subplacenta is known to be responsive to the amount of oxygen available, and its area is increased when oxygen is restricted [46]. The fact that we have observed vesicles and secretory granules in addition to microvilli in the subplacenta cells of the paca and the guinea pig also suggests that there may be local effects of secreted products within the placenta itself, suggestive of paracrine effects.

In addition to sub-cellular specializations, the subplacenta of several species examined here (capybara, paca, agouti and rock cavy) presents a distinct pattern of vascularization, typical of endocrinal glands. Different aspects including the central vasculature of the labyrinth and junctional zones, and subplacental vasculature, were comparable with small capillaries, around larger vessels, where it was possible to identify sinusal dilatations and anastomotic channels, like those described previously [1]. Furthermore, others reported that the intercellular space was marked by the presence of microvilli as evidence of communication between the syncytium and the cytotrophoblast that make up the subplacenta in the cane rat [47]. While their content remains unknown, there is a temporal concordance between the appearance of these secretory granules in the subplacenta and the increase in progesterone binding globulins during gestation. In addition, the peroxisomes found in the subplacenta are consistent with lipid metabolism. These organelles contain enzymes related to the beta-oxidation of fatty acids. Peroxisomes breakdown fatty acids into acetyl-CoA, which supports the biosynthesis of cholesterol, the ultimate precursor of all steroid hormones [48]. Burgess and Tam [34] reported that lipid droplets which were first apparent in the spongy zone and other areas of the placenta after day 25 of gestation were consistent with steroidogenic activity in these cells. Mitochondria, especially those derived from syncytiotrophoblast, are known to have the capacity to initiate steroid hormone synthesis, being the sub-cellular site of expression of cytochrome P450 cholesterol side chain cleavage [49, 50].

More direct evidence for endocrine function was provided by the detection of steroid hormones in subplacental tissue and the expression of steroidogenic enzymes including 17β-HSD and 3β-HSD by immuno-histochemistry which is consistent with the synthesis of progesterone and of testosterone and estradiol. The catalytic activity of 17β-HSD is required for the reduction of DHEA to androstenediol, androstenedione to testosterone and estrone to estradiol. The presence of progesterone in subplacental tissue is also consistent with expression of 3β-HSD and the oxidation of pregnenolone and 17α-hydroxypregnenolone to progesterone and 17α-hydroxyprogesterone, respectively [51, 52]. The failure to detect aromatase labeling is consistent with the low amount of estradiol found in the tissues compared to progesterone and testosterone. This is also consistent with the low levels of expression of aromatase compared with cytochrome P450 17α-hydroxylase/1720-lyase in ovarian [53] and testicular tissues [54]. Finally, immunohistochemistry is not a very sensitive technique and low levels of enzyme activity together with poor cross-reactivity of antisera across species can present challenges.

## Conclusions

The subplacenta does not differ greatly in terms of the structural aspects among the hystricomorphs investigated, and it is has potential for hormonal secretion during the early stages of pregnancy based on evidence consistent with steroidogenic enzyme activity.

## Data Availability

The data that support the findings of this study are openly available. If necessary, contact Maria Angelica Miglino (miglino@usp.br).

## Abbreviations

PBS - phosphate: Buffered saline
IM: Immunohistochemistry
mM: Millimolar
BSA: Bovine serum albumin
17β-HSD: 17β-hydroxysteroid dehydrogenase
3β-HSD: 3β-hydroxysteroid dehydrogenase
P450: Aromatase cytochrome
SEM: Scanning Electron Microscopy
EIA: Enzyme-immunoassays
DHEA: Dehydroepiandrosterone
